# Based on network pharmacology and molecular docking to predict the mechanism of TMDZ capsule in the treatment of IS

**DOI:** 10.1097/MD.0000000000034424

**Published:** 2023-07-28

**Authors:** Fengjiao Yang, Yun Gu, Ya Yan, Guangming Wang

**Affiliations:** a School of Clinical Medicine, Dali University, Dali, PR China; b College of Pharmacy, Dali University, Dali, PR China.

**Keywords:** IS, mechanism of action, molecular docking, network pharmacology, TMDZ capsules

## Abstract

Ischemic stroke (IS) is the most common cardiovascular and cerebrovascular disease in clinic. Qiangli Tianma Duzhong Capsule (TMDZ capsule) has significant therapeutic effect to IS. Therefore, it is great significance to explore the mechanism of action of TMDZ capsules in the treatment of IS.

**Methods:**

The potential active components and possible targets of TMDZ capsule were obtained from TCMSP and The Encyclopedia of Traditional Chinese Medicine databases. IS related targets were collected by Genecard database, OMIM database, TTD database and DisGeNET database. The common target network of drug-diseases was constructed using Cytoscape for visualization analysis. Potential mechanisms were identified through enrichment analysis of gene Ontology (GO) and Kyoto Encyclopedia of Genes and Genomes (KEGG). Three key targets (ALB, TNF, and INS) were selected from the key networks with high correlation scores in PPI for molecular docking, through molecular docking, the interaction between target and protein is visualized.

**Results:**

59 active components and 648 targets of TMDZ capsules and 2286 targets of IS were obtained through database mining. Compound-target network is constructed with 117 nodes and 1185 edges. GO and KEGG suggest that lipids and atherosclerosis, fluid shear forces and atherosclerosis, neurodegenerative pathways – multiple diseases and blood circulation play important roles in the treatment of IS.

**Conclusions:**

This study reveals the molecular mechanism of TMDZ capsules in the treatment of IS by integrating molecular docking with a network pharmacological strategy, which not only confirmed the clinical efficacy of TMDZ capsule, but also laid the foundation for further experimental research.

## 1. Introduction

Stroke is the most common cardiovascular and cerebrovascular disease in clinic^[1]^ and also known as cerebrovascular accident, it is the second leading cause of death and the third leading cause of disability in the world.^[2]^ Its 2 major categories are ischemic stroke (IS) and hemorrhagic stroke.^[3]^ About 18 million people suffered from IS in 2020, with two-thirds of IS occurring in people over 65 years of age,^[4,5]^ and South Asians are higher risk of developing IS particularly, accounting for 40% of global IS deaths, it imposes an enormous burden on health care systems worldwide. IS accounts for 70% to 80% of stroke, and the research on effective treatment of IS is one of the hot spots of stroke at present.^[6,7]^

IS is characterized by early absence of obvious symptoms and high recurrence rate, it is easy to develop into cerebral infarction with the gradual aggravation of the disease.^[8]^ With the clinical gradual attention of traditional Chinese medicine (TCM) treatment of diseases and Chinese medicine has also made positive progress in the treatment of cardiovascular and cerebrovascular diseases, so we should actively adopt the method of integrated traditional Chinese and western medicine for clinical treatment.^[2,9]^ The common pathogenic factors of IS are arterial thrombosis and venous thrombosis, which are distinguished by embolism of attached blood vessels, arterial thrombosis is associated with a high risk of IS. In view of the behavioral disorders caused by IS, TCM put forward the concept of “qi deficiency and blood stasis.”^[6,10,11]^ From the TCM for the treatment of IS, it was found that the Qiangli Tianma Duzhong Capsule (TMDZ capsule) contained part of the TCM for the treatment of IS, According to the investigation, the TMDZ capsule with TCM ingredients has obvious clinical efficacy in the treatment of cardiovascular and cerebrovascular diseases.^[6]^

**Figure 1.**
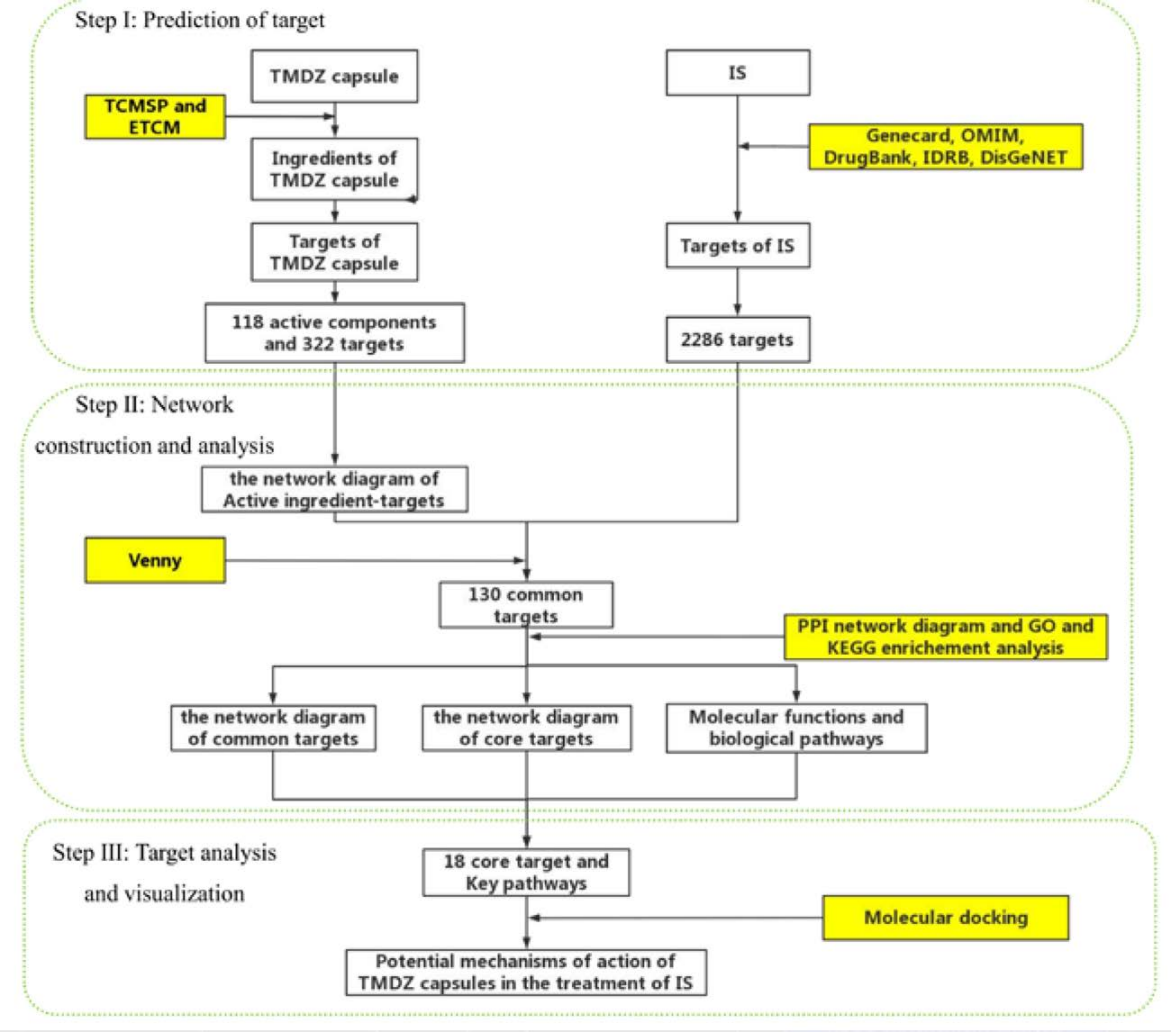
Work flow chart of this study. ETCM = The Encyclopedia of Traditional Chinese Medicine, GO = gene ontology, IS = ischemic stroke, KEGG = Kyoto Encyclopedia of Genes and Genomes, PPI = protein-protein interaction, TCMSP = Traditional Chinese Medicine Systems Pharmacology Database and Analysis Platform, TMDZ capsule = Qiangli Tianma Duzhong Capsule.

At present, TMDZ capsule has been registered in the national medical insurance, in clinical practice, it is mainly used for the symptoms of meridian pain, limb numbness, inconvenient walking, waist and leg pain, headache and dizziness caused by stroke. TMDZ capsules can improve microcirculation, slow heart rate, prolong ejection time, increase cerebral blood flow, reduce myocardial oxygen consumption, have good sedative and hypnotic effects, and reduce sympathetic nerve excitability, these effects have certain therapeutic on related symptoms caused by IS.^[12]^ TMDZ capsule is a multi-target drug with good efficacy in the treatment of IS, the network pharmacology method can explain the characteristics of “multi-component and multi-target” of TMDZ capsule, which is consistent with the complex etiology and multi target treatment concept of IS.^[13,14]^ In this study, network pharmacology was adopted to predict the active ingredients, targets and pathways of TMDZ capsules, the specific steps are shown in Figure 1. Finally, key targets of TMDZ capsules in the treatment of IS were located in the integrated gene expression dataset, which could evaluate the corresponding relationship and explain the mechanism of action of TMDZ capsules in the treatment of IS. It could provide a new strategy in the treatment of IS.

## 2. Materials and methods

### 2.1. Screening of active ingredients of TMDZ capsules

The Encyclopedia of Traditional Chinese Medicine (ETCM; http://www.tcmip.cn/ETCM/index.php/Home/Index/)^[15]^ and Traditional Chinese Medicine Systems Pharmacology Database and Analysis Platform (TCMSP; https://tcmspw.com/tcmsp.php)^[16]^ can provide comprehensive information, which is ingredients, targets and pharmacokinetic characteristics of commonly used TCM herbs and herbal formulas. In this study, we accessed the ETCM and TCMSP to predict the ADME-related properties of TMDZ capsules by searching for the chemical name “Gastrodia elata, Eucommia ulmoides (salt), Radix Aconiti Kusnezoffii, Aconitum carmichaelii Debx, Radix Angelicae Pubescentis, Ligusticum, scrophularia ningpoensis, Angelica sinensis, rehmannia, Cyathula officinalis, Viscum coloratum and Notopterygium incisum,” which can collect components, targets and other relevant properties of TMDZ capsules.

Because the use of the ETCM and TCMSP to predict the potential compounds and target of TMDZ capsules has limitations, we searched the extensive literature through the Wanfang database, CNKI, Chaoxing, PubMed and other databases to supplement the relevant data. Then targets were entered into the Uniport database for target normalization.

### 2.2. Collection of target proteins of IS

IS targets are collected from different databases in preparation for subsequent analysis. Keywords “ischemic stroke” for Genecard database (https://www.genecards.org/),^[17]^ Online Mendelian Inheritance in Man database (http://www.omim.org),^[18]^ DrugBank database (https://www.drugbank.ca/)^[19]^ and the therapeutic target database (http://db.idrblab.net/ttd/),^[20]^ the results of the 5 databases was summarized, duplicates were removed, finally, IS-related targets were obtained.

All targets were standardized using the Uniprot database and the species should be selected as “Homo snpiens” when using the Uniprot database for target and Gene name conversion.

### 2.3. Screening of common targets of drug components and diseases

The core targets of TMDZ capsules in the treatment of IS were obtained by intersecting the targets of the active chemical components in TMDZ capsules with the genes related to IS disease and the Venn diagram was prepared, which requires the use of the Venny online platform (https://bioinfogp.cnb.csic.es/tools/venny_old/).^[21]^

### 2.4. The network diagram of “active ingredient-target”

In order to elucidate the relationship between the active chemical constituents and targets, the composition-target diagram of drug was constructed by Cytoscape3.8.2. Important network topological parameters of the compound and related targets were calculated, such as degree, intermediate centrality, and proximity centrality. the larger the node and node connectivity, which related to the more functions in the network diagram.

### 2.5. Construction of PPI network diagram and screening of core targets of TMDZ capsules in the treatment of IS

Protein-protein interaction (PPI) network diagrams are constructed to illustrate the interactions of proteins and interactions between proteins and other molecules, which can predict intracellular effects. The core target of TMDZ capsule treatment IS entered into the String online platform (http://string-db.org/)^[22]^ for PPI, which can explain the therapeutic mechanism of action of TMDZ capsule more comprehensively. In order to meet the accuracy and quantity requirements of PPI, the confidence score was selected to be greater than 0.4, the species was limited to *Homo sapiens*, then PPI data was obtained. The results are saved in tsv format. PPI results are imported into Cytoscape3.8.2 software to draw the interactive network, then core targets were selected by double degree value.

### 2.6. Analysis of target function and pathway enrichment in the treatment of IS with TMDZ capsule

To further clarify relevant functions and pathways, gene ontology (GO) functional enrichment analysis and Kyoto Encyclopedia of Genes and Genomes (KEGG) pathway enrichment analysis were carried out by the metascape platform (http://metascape.prg/gp/index.html),^[23]^ results were obtained with human was defined as the species. After getting the results, these results are visualized as GO term figure and bubble chart based on bio-informatics platform (www.bio-informatics.com.cn). metascape platform is a powerful gene functional database, which can provide comprehensive functional annotations of pathways, including biological processes, cellular component and molecular functions.

### 2.7. Molecular docking validation

Degree of nodes is a key indicator to describe network nodes. The top 3 core targets of degree values (ALB, TNF, and INS) were selected from PPI networks. The corresponding chemical components of p-hydroxybenzaldehyde, carotenoside and palmitic acid were found, then Molecular docking of the target and the corresponding chemical components was performed. The protein structure of the key target was obtained from the PDB database (https://www.rcsb.org/),^[24]^ the chemical structure of the 3 components was drawn by ChemDraw18.1. The prepared protein structure and molecular structure were imported into PyMOL to remove water molecules and small molecule ligands, the AutoDockTools software was used to convert the protein and drug components into pdqt format files and identify active pockets, finally, molecular docking was performed by vina software. The greater the absolute value of binding energy, the stronger the binding ability of the compound and the protein target.

## 3. Result

### 3.1. Active compounds and putative targets of TMDZ capsule

The active compounds of the 12 TCM were obtained in TMDZ capsules from the ETCM database and TCMSP database. A total of 118 active components were obtained, 12 components from Gastrodia elata, 28 components from Eucommia ulmoides, 8 components from Radix Aconiti Kusnezoffii, 21 components from Aconitum carmichaelii Debx, 9 components from Radix Angelicae Pubescentis, 1 component from Ligusticum, 9 components from scrophularia ningpoensis, 2 components from Angelica sinensis, 2 components from rehmannia, 4 components from Cyathula officinalis, 7 components from Viscum coloratum, and 15 components from Notopterygium incisum, there were 11 duplicate compounds (19 duplicate values), 99 active compounds were obtained after removing the duplicate values. A total of 648 genes were identified from the ETCM and TCMSP databases, 345 targets from Gastrodia elata, 248 targets from Eucommia ulmoides, 8 targets from Radix Aconiti Kusnezoffii, 17 targets from Aconitum carmichaelii Debx, 35 targets from Radix Angelicae Pubescentis, 4 targets from Ligusticum, 23 targets from scrophularia ningpoensis, 62 targets from Angelica sinensis, 93 targets from Cyathula officinalis, 61 targets from Viscum coloratum and 38 targets from Notopterygium incisum, excluding 326 common genes, finally, 322 genes were identified.

### 3.2. Putative targets of IS

A total of 2334 targets were identified, 2144 targets were identified in Genecard database, 129 targets were identified in OMIM database, 61 targets were identified in DrugBank database and 0 targets were identified in the therapeutic target database database. 2286 targets were defined after removing 48 duplicate targets.

### 3.3. Analysis of “active ingredient-target” network diagram

**Figure 2.**
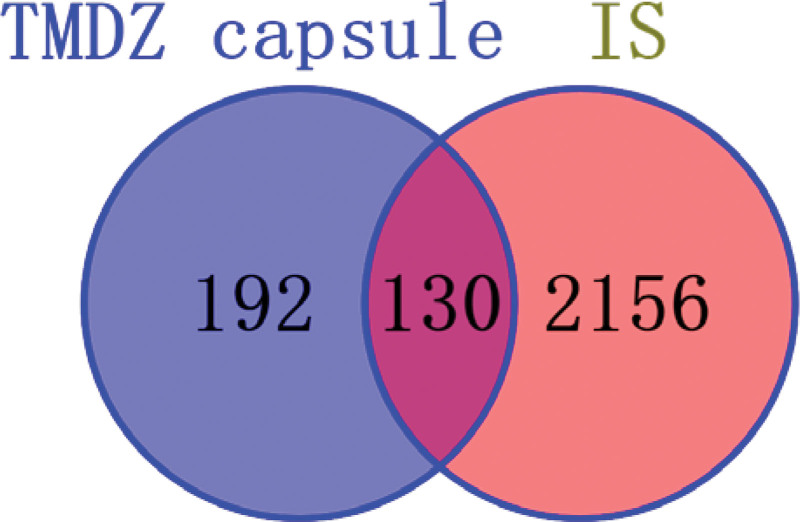
Venn diagram of drug and disease common targets. IS = ischemic stroke, TMDZ capsule = Qiangli Tianma Duzhong Capsule.

**Figure 3.**
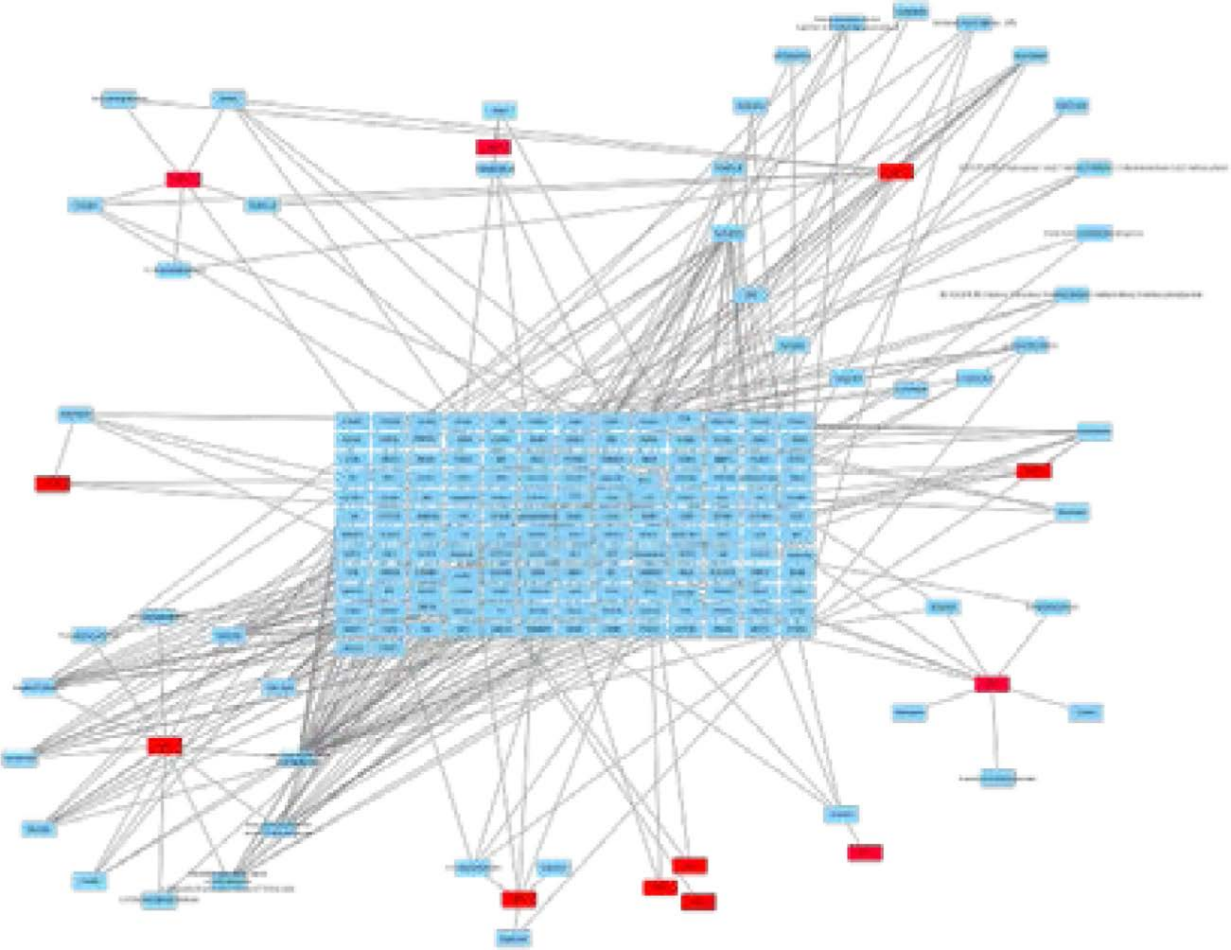
“Active ingredient-target” network diagram of TMDZ capsules. TMDZ capsule = Qiangli Tianma Duzhong Capsule.

Through Venny online platform, 130 common targets of drugs and diseases were obtained, as shown in Figure 2. “active ingredient-target” network diagram of TMDZ capsules is shown in Figure 3. which can obtain 127 nodes and 1185 edges. it shows that Gastrodia elata and eucommia ulmoides have more relevant components and targets, while Cyathula officinalis and Radix Aconiti Kusnezoffii have less relevant components and targets. the node of higher degree can connect more edge, which indicate that the node is important in the network, so CASP3 is associated with a variety of herbs in TMDZ capsules, which indicate that CASP3 is important.

### 3.4. Construction of PPI network diagram and screening of core targets

**Figure 4.**
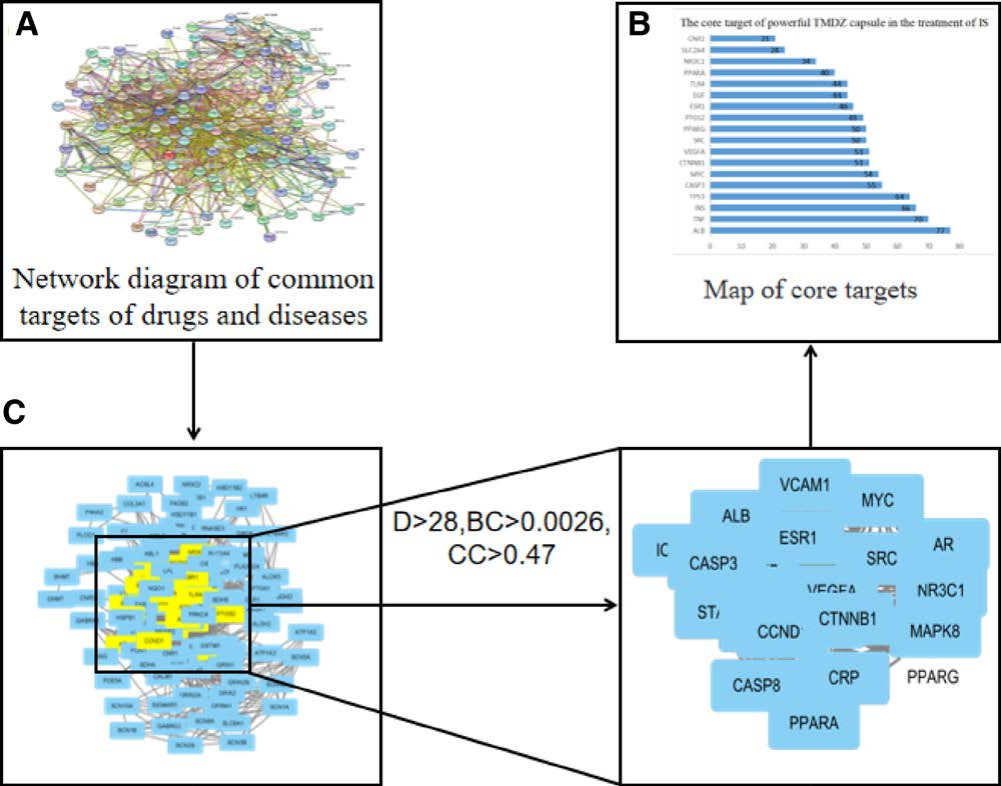
Screening process for core targets of TMDZ capsules in the treatment of IS. IS = ischemic stroke, TMDZ capsule = Qiangli Tianma Duzhong Capsule.

Protein interaction relationship data and protein interaction network diagram were obtained, as shown in Figure 3. 127 nodes and 1185 edges were obtained. 25 nodes and 268 edges were obtained after screening key targets. 18 key targets were obtained through PPI network screening, the result is shown in Figure 4. In the PPI network, the darker the color and the larger the node area, which indicate the node is more important, among them, the top 10 targets with high degree value were albumin (ALB), tumor necrosis factor (TNF), insulin (INS), cell tumor antigen p53 (TP53), caspase 3 (CASP3), Myelocytomatosis (MYC), Catenin Beta-1 (CTNNB1), Vascular Endothelial growth factor A (VEGFA), sparse representation-based classifier (SRC), peroxisome proliferative activated receptor γ (PPARG), prostaglandin proliferative peroxidase synthase 2 (PTGS2).

### 3.5. Functional and pathway enrichment analysis of key targets

**Figure 5.**
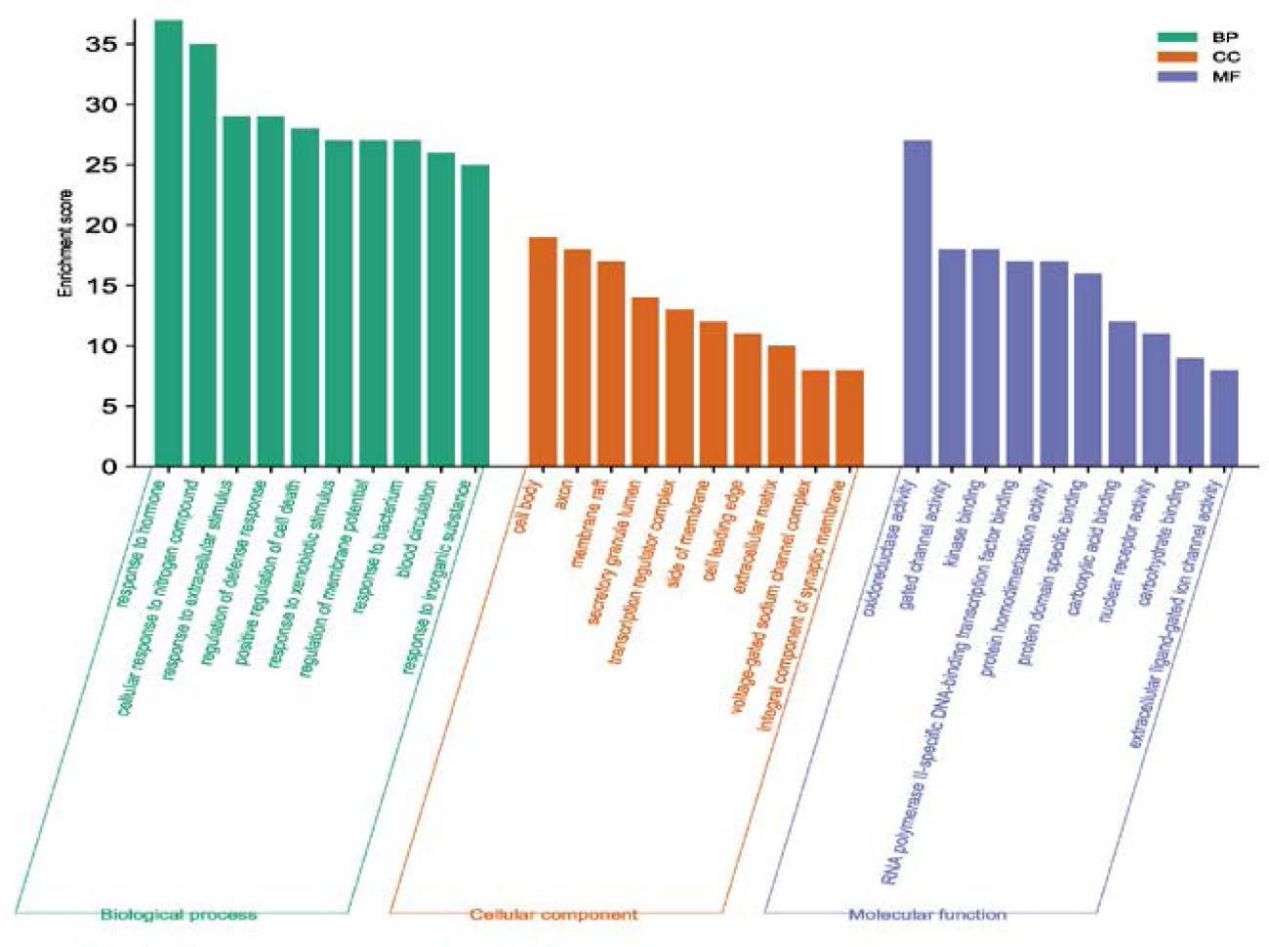
Enrichement GO term. GO = gene ontology.

**Figure 6.**
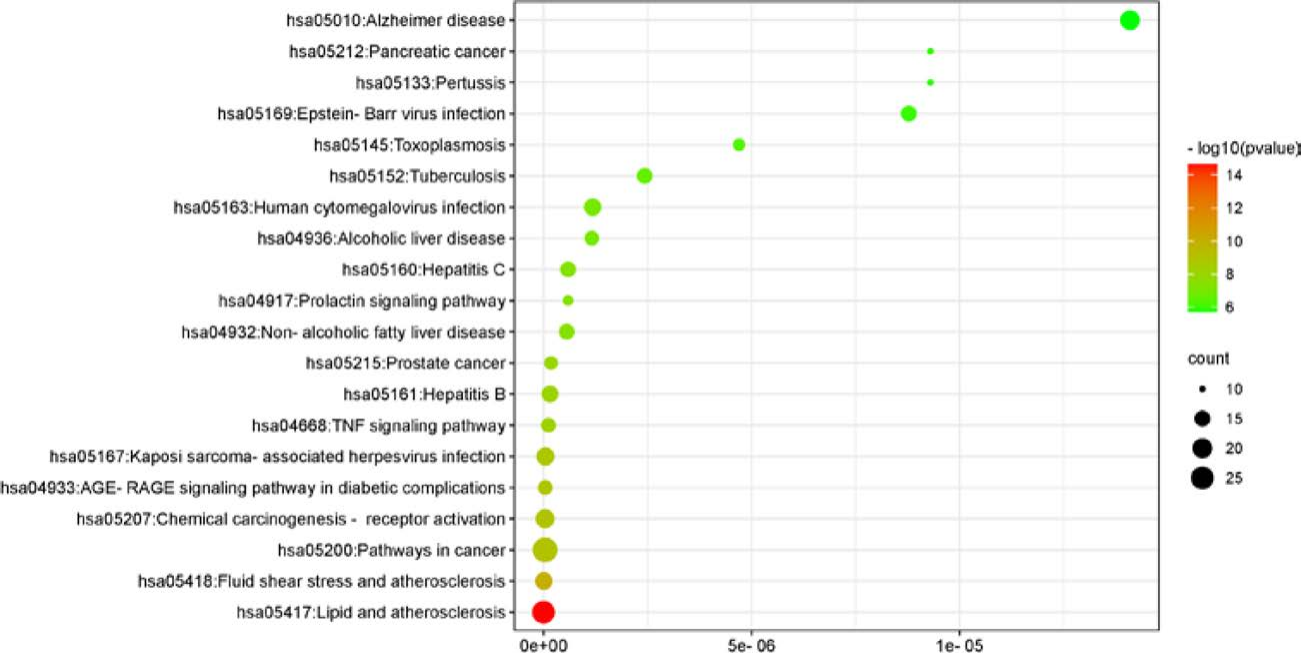
Dot bubble of KEGG pathways. KEGG = Kyoto Encyclopedia of Genes and Genomes.

In order to further explore the mechanism of action of TMDZ capsule treatment of IS, potential therapeutic targets were analyzed for GO and KEGG enrichment (the top 10 results of each analysis are shown in Table 1). GO functional enrichment analysis showed that GO entries included 470 biological processes (BP), 72 cellular components (CC), and 133 molecular functions (MF). The first 10 entries of −lgP values of BP, CC and MF are shown in Figure 5. A total of 141 KEGG pathway enrichment analyses were obtained. According to the −lgP value, top 10 items is shown in Table 1 and the bubble plot of top 20 items is shown in Figure 6.

**Table 1:** Xxxx.

Category	Serial number	Description	Log P	Log (q value)	Gene
Composition of cells	GO:0001518	Voltage-gated sodium channel complex	−14.6515482	−11.362	SCN1A,SCN1B,SCN2B,SCN4B,SCN5A,SCN8A,SCN10A,SCN3B
	GO:1902495	Transmembrane transporter complex	−13.28462243	−10.474	ATP1A2,ATP1A3,CALM1,GABRA2,GABRG2,GRIA2,GRIN1,GRIN2A,GRIN2B,HTR3A,SCN1A,SCN1B,SCN2B,SCN4B,SCN5A,SCN8A,SCN10A,SCN3B
	GO:0045121	Membrane raft	−13.18303848	−10.474	ATP1A2,CASP3,CASP8,CNR1,CTNNB1,CTSD,HK1,ICAM1,OLR1,OPRM1,FURIN,PTGS2,SCN5A,SELE,SLC2A4,SRC,TNF
	GO:0098857	Membrane microdomain	−13.16140026	−10.474	ATP1A2,CASP3,CASP8,CNR1,CTNNB1,CTSD,HK1,ICAM1,OLR1,OPRM1,FURIN,PTGS2,SCN5A,SELE,SLC2A4,SRC,TNF
	GO:0034706	Sodium channel complex	−12.85763825	−10.335	SCN1A,SCN1B,SCN2B,SCN4B,SCN5A,SCN8A,SCN10A,SCN3B
	GO:1990351	Transporter complex	−12.84669184	−10.335	ATP1A2,ATP1A3,CALM1,GABRA2,GABRG2,GRIA2,GRIN1,GRIN2A,GRIN2B,HTR3A,SCN1A,SCN1B,SCN2B,SCN4B,SCN5A,SCN8A,SCN10A,SCN3B
	GO:0034702	Ion channel complex	−12.71126152	−10.267	CALM1,GABRA2,GABRG2,GRIA2,GRIN1,GRIN2A,GRIN2B,HTR3A,SCN1A,SCN1B,SCN2B,SCN4B,SCN5A,SCN8A,SCN10A,SCN3B
	GO:0034703	Cation channel complex	−12.09016686	−9.704	CALM1,GRIA2,GRIN1,GRIN2A,GRIN2B,HTR3A,SCN1A,SCN1B,SCN2B,SCN4B,SCN5A,SCN8A,SCN10A,SCN3B
	GO:0098797	Plasma membrane protein complex	−11.4175697	−9.082	ATP1A2,ATP1A3,CALM1,CASP3,CASP8,CTNNB1,GRIA2,GRIN1,GRIN2A,GRIN2B,HTR3A,PRKCA,SCN1A,SCN1B,SCN2B,SCN4B,SCN5A,SCN8A,SCN10A,VCAM1,SCN3B
	GO:0044297	Cell body	−10.55529632	−8.266	ABL1,ACTA1,ANG,ATP1A2,ATP1A3,CASP3,CASP8,CNR2,NQO1,GABRA2,GRIA2,OPRM1,S100B,SCN1A,SCN1B,SLC8A1,TNF,TRPV1,GABRG2,GRIN1,GRIN2A,MAPK8,STAT1
Molecular Function bioprocess	GO:0016491	Oxidoreductase activity	−17.00569652	−13.321	ALDH2,ALOX5,ASPH,CYP1B1,CYP2B6,CYP2C8,CYP3A4,NQO1,GSTP1,HBA1,HBB,HSD11B1,HSD11B2,IDH1,IDH2,FADS1,PLOD1,PTGS1,PTGS2,SDHA,SDHB,SDHC,SDHD,TYR,VCAM1,P4HA2,FADS2
	GO:0004879	Nuclear receptor activity	−15.49789141	−12.290	AR,ESR1,ESR2,NR3C1,HNF4A,NR3C2,PPARA,PPARD,PPARG,SREBF1,VDR
	GO:0098531	Ligand-activated transcription factor activity	−15.49789141	−12.290	AR,ESR1,ESR2,NR3C1,HNF4A,NR3C2,PPARA,PPARD,PPARG,SREBF1,VDR
	GO:0008289	Lipid binding	−14.17320406	−11.090	ALB,AR,ATP1A2,ATP1A3,CYP3A4,ESR1,ESR2,NR3C1,GSTP1,HNF4A,HSD11B1,HSD11B2,LPL,LTF,NR3C2,PLA2G2A,PON1,PPARA,PPARD,PPARG,RNASE3,TLR4,VDR,TRPV1,LY96
	GO:0022836	Gated channel activity	−14.05497534	−11.069	CNR1,GABRA2,GABRG2,GRIA2,GRIN1,GRIN2A,GRIN2B,HTR3A,OPRM1,SCN1A,SCN1B,SCN2B,SCN4B,SCN5A,SCN8A,SCN10A,TRPV1,SCN3B,BCL2,ATP1A2,ATP1A3,SLC8A1,CALM1,CTNNB1,SRC
	GO:0005248	Voltage-gated sodium channel activity	−13.18170533	−10.275	SCN1A,SCN1B,SCN2B,SCN4B,SCN5A,SCN8A,SCN10A,SCN3B
	GO:0061629	RNA polymerase II-specific DNA-binding transcription factor binding	−12.72310743	−9.942	PARP1,AR,CTNNB1,ESR1,HNF4A,HSPB1,NFE2L2,PPARA,PPARD,PPARG,RB1,RELA,SRC,STAT1,TP53,VDR,HDAC9
	GO:0140297	DNA-binding transcription factor binding	−12.71707951	−9.942	PARP1,AR,BCL2,CTNNB1,ESR1,HNF4A,HSPB1,MYC,NFE2L2,PPARA,PPARD,PPARG,RB1,RELA,SRC,STAT1,TP53,VDR,HDAC9
	GO:0005244	Voltage-gated ion channel activity	−12.62735125	−9.942	CNR1,GRIN1,GRIN2A,GRIN2B,OPRM1,SCN1A,SCN1B,SCN2B,SCN4B,SCN5A,SCN8A,SCN10A,TRPV1,SCN3B
	GO:0022832	Voltage-gated channel activity	−12.62735125	−9.942	CNR1,GRIN1,GRIN2A,GRIN2B,OPRM1,SCN1A,SCN1B,SCN2B,SCN4B,SCN5A,SCN8A,SCN10A,TRPV1,SCN3B
	GO:1901699	Cellular response to nitrogen compound	−29.28343878	−25.374	ABL1,ACHE,ACTA1,PARP1,ATP1A3,CASP3,CASP7,COL3A1,CTNNB1,CYP1B1,GABRG2,GCK,HTR3A,ICAM1,IFNB1,INS,MMP3,MYC,NFE2L2,NFKB1,OPRM1,PPARG,PTGS2,RB1,RELA,SLC2A4,SLC8A1,SRC,SREBF1,STAT1,TLR4,TNF,TP53,VCAM1,TRPV1,HDAC9
	GO:0009725	Response to hormone	−29.26024553	−25.374	ACTA1,PARP1,ANG,AR,ATP1A2,ATP1A3,CCND1,BCL2,CASP3,CASP9,COL3A1,CYP1B1,NQO1,ESR1,ESR2,F7,GCK,NR3C1,HSD11B2,IDH1,INS,NR3C2,MYC,NFE2L2,NFKB1,PPARA,PPARD,PPARG,PTGS2,RB1,RELA,SLC2A4,SRC,SREBF1,STAT1,TNF,TRPV1,HDAC9
	GO:0071417	Cellular response to organonitrogen compound	−26.9012772	−23.191	ABL1,ACTA1,PARP1,ATP1A3,CASP3,CASP7,COL3A1,CTNNB1,CYP1B1,GABRG2,GCK,HTR3A,ICAM1,INS,MYC,NFE2L2,NFKB1,OPRM1,PPARG,PTGS2,RB1,RELA,SLC2A4,SLC8A1,SRC,SREBF1,STAT1,TLR4,TNF,TP53,VCAM1,TRPV1,HDAC9
	GO:0042391	Regulation of membrane potential	−24.42910269	−20.843	ABL1,PARP1,ATP1A2,ATP1A3,BCL2,CALM1,CNR1,CNR2,GABRA2,GABRG2,GRIN1,GRIN2A,GRIN2B,HTR3A,MYC,OPRM1,SCN1A,SCN1B,SCN2B,SCN4B,SCN5A,SCN8A,SCN10A,SLC8A1,SRC,TNF,TRPV1,SCN3B
	GO:0009991	Response to extracellular stimulus	−24.30715588	−20.818	ACTA1,ALB,CCND1,BCL2,CNR1,CYP1B1,NQO1,F7,HSD11B2,FADS1,LPL,NFE2L2,OPRM1,PON1,PPARA,PPARD,PPARG,MAPK8,PTGS2,SLC8A1,SRC,SREBF1,STAT1,TNF,TP53,TYR,VCAM1,VDR,TRPV1
	GO:1901652	Response to peptide	−24.01614469	−20.607	PARP1,ATP1A3,COL3A1,CYP1B1,F7,GCK,HSD11B2,ICAM1,INS,MMP3,MYC,NFE2L2,NFKB1,PPARA,PPARG,PTGS2,RB1,RELA,SLC2A4,SRC,SREBF1,STAT1,TLR4,TNF,TP53,VCAM1,TRPV1,HDAC9
	GO:0031667	Response to nutrient levels	−23.88187814	−20.539	ALB,CCND1,BCL2,CNR1,CYP1B1,NQO1,F7,HSD11B2,FADS1,LPL,NFE2L2,OPRM1,PON1,PPARA,PPARD,PPARG,MAPK8,PTGS2,SLC8A1,SRC,SREBF1,STAT1,TNF,TP53,TYR,VCAM1,VDR,TRPV1
	GO:0009410	Response to xenobiotic stimulus	−23.81868471	−20.534	ABL1,CCND1,BCL2,CASP3,CTNNB1,CYP1B1,CYP2B6,CYP2C8,CYP3A4,NQO1,GRIN1,GRIN2A,GSTM1,GSTP1,HNF4A,HSD11B2,LPL,MYC,NFE2L2,PTGS2,RB1,SLC8A1,SRC,SREBF1,STAT1,TNF,TP53
	GO:0071407	Cellular response to organic cyclic compound	−23.65880444	−20.425	ABL1,AR,ATP1A2,ATP1A3,CASP3,CASP7,CASP8,CASP9,CTNNB1,CYP1B1,ESR1,ESR2,GABRG2,NR3C1,HTR3A,IFNB1,NR3C2,MYC,NFKB1,OPRM1,PPARA,PPARD,PTGS2,SLC8A1,SRC,STAT1,TNF,VDR,TRPV1
	GO:0008015	Blood circulation	−22.36751282	−19.180	ABL1,AR,ATP1A2,CNR1,CRP,HBB,HSD11B2,INS,OLR1,PPARA,PPARD,PPARG,PTGS1,PTGS2,SCN1A,SCN1B,SCN2B,SCN4B,SCN5A,SLC8A1,SRC,STAT1,TNF,VEGFA,TRPV1,SCN3B,SLC2A4
KEGG pathway	hsa05417	Lipid and atherosclerosis	−28.00580733	−25.467	BCL2,CALM1,CASP3,CASP7,CASP8,CASP9,CYP2B6,CYP2C8,ICAM1,IFNB1,MMP3,NFE2L2,NFKB1,OLR1,PPARG,PRKCA,MAPK8,RELA,SELE,SRC,TLR4,TNF,TP53,VCAM1,LY96
	hsa05200	Pathways in cancer	−23.04587648	−20.808	ABL1,AR,CCND1,BCL2,CALM1,CASP3,CASP7,CASP8,CASP9,CTNNB1,NQO1,EGF,ESR1,ESR2,GSTM1,GSTP1,MYC,NFE2L2,NFKB1,PPARD,PPARG,PRKCA,MAPK8,PTGS2,RB1,RELA,STAT1,TP53,VEGFA
	hsa05418	Fluid shear stress and atherosclerosis	−19.44605976	−17.384	BCL2,CALM1,CTNNB1,NQO1,GSTM1,GSTP1,ICAM1,NFE2L2,NFKB1,MAPK8,RELA,SELE,SRC,TNF,TP53,VCAM1,VEGFA
	hsa05207	Chemical carcinogenesis - receptor activation	−19.09237525	−17.155	AR,CCND1,BCL2,CYP1B1,CYP2B6,CYP3A4,EGF,ESR1,ESR2,GSTM1,MYC,NFKB1,PPARA,PRKCA,RB1,RELA,SRC,VDR,VEGFA
	hsa05167	Kaposi sarcoma-associated herpesvirus infection	−18.37109198	−16.531	CCND1,CALM1,CASP3,CASP8,CASP9,CTNNB1,ICAM1,IFNB1,MYC,NFKB1,MAPK8,PTGS2,RB1,RELA,SRC,STAT1,TP53,VEGFA
	hsa04933	AGE-RAGE signaling pathway in diabetic complications	−16.92728945	−15.166	CCND1,BCL2,CASP3,COL3A1,ICAM1,NFKB1,PRKCA,MAPK8,RELA,SELE,STAT1,TNF,VCAM1,VEGFA
	hsa05161	Hepatitis B	−16.79964648	−15.106	BCL2,CASP3,CASP8,CASP9,IFNB1,MYC,NFKB1,PRKCA,MAPK8,RB1,RELA,SRC,STAT1,TLR4,TNF,TP53
	hsa04668	TNF signaling pathway	−16.21065351	−14.575	CASP3,CASP7,CASP8,ICAM1,IFNB1,IRF1,MMP3,NFKB1,MAPK8,PTGS2,RELA,SELE,TNF,VCAM1
	hsa05022	Pathways of neurodegeneration – multiple diseases	−15.90502067	−14.320	BCL2,CALM1,CASP3,CASP7,CASP8,CASP9,CTNNB1,GRIA2,GRIN1,GRIN2A,GRIN2B,NFKB1,PRKCA,MAPK8,PTGS2,RELA,SDHA,SDHB,SDHC,SDHD,TNF,SIGMAR1
	hsa05163	Human cytomegalovirus infection	−15.84931123	−14.310	CCND1,CALM1,CASP3,CASP8,CASP9,CTNNB1,IFNB1,MYC,NFKB1,PRKCA,PTGS2,RB1,RELA,SRC,TNF,TP53,VEGFA
	hsa04932	Nonalcoholic fatty liver disease	−15.6307341	−14.133	CASP3,CASP7,CASP8,INS,NFKB1,PPARA,PPARG,MAPK8,RELA,SDHA,SDHB,SDHC,SDHD,SREBF1,TNF
	hsa05160	Hepatitis C	−15.54633574	−14.086	CCND1,CASP3,CASP8,CASP9,CTNNB1,EGF,IFNB1,MYC,NFKB1,PPARA,RB1,RELA,STAT1,TNF,TP53
	hsa05215	Prostate cancer	−15.48451406	−14.059	AR,CCND1,BCL2,CASP9,CTNNB1,EGF,GSTP1,INS,MMP3,NFKB1,RB1,RELA,TP53
	hsa05010	Alzheimer disease	−15.44944154	−14.056	CALM1,CASP3,CASP7,CASP8,CASP9,CTNNB1,GRIN1,GRIN2A,GRIN2B,INS,LPL,NFKB1,MAPK8,PTGS2,RELA,SDHA,SDHB,SDHC,SDHD,TNF
	hsa04936	Alcoholic liver disease	−14.72853002	−13.366	ALDH2,CCND1,CASP3,CASP8,CTNNB1,IFNB1,NFKB1,PPARA,MAPK8,RELA,SREBF1,TLR4,TNF,LY96
	hsa05152	hsa05152	−14.65221072	−13.317	BCL2,CALM1,CASP3,CASP8,CASP9,CTSD,IFNB1,NFKB1,MAPK8,RELA,SRC,STAT1,TLR4,TNF,VDR
	hsa04917	Prolactin signaling pathway	−13.96753461	−12.659	CCND1,ESR1,ESR2,GCK,INS,IRF1,NFKB1,MAPK8,RELA,SRC,STAT1
	hsa05169	Epstein-Barr virus infection	−13.90627231	−12.622	CCND1,BCL2,CASP3,CASP8,CASP9,ICAM1,IFNB1,MYC,NFKB1,MAPK8,RB1,RELA,STAT1,TNF,TP53
	hsa05208	Chemical carcinogenesis - reactive oxygen species	−13.2724625	−12.012	ABL1,CYP1B1,NQO1,EGF,GSTM1,NFE2L2,NFKB1,MAPK8,RELA,SDHA,SDHB,SDHC,SDHD,SRC,VEGFA
	hsa05145	Toxoplasmosis	−13.12584937	−11.888	ALOX5,BCL2,CASP3,CASP8,CASP9,NFKB1,MAPK8,RELA,STAT1,TLR4,TNF,LY96

GO = gene ontology, KEGG = Kyoto Encyclopedia of Genes and Genomes.

Through GO functional enrichment analysis and KEGG pathway analysis, ion channel complex, lipid binding, blood circulation, lipids and atherosclerosis, fluid shear stress and atherosclerosis, pathways of neurodegeneration multiple diseases and other processes were related to the treatment of IS with TMDZ capsule. Through the above process, we can speculate the mechanism of action of TMDZ capsule in the treatment of IS.

### 3.6. Molecular docking of active compounds

**Figure 7.**
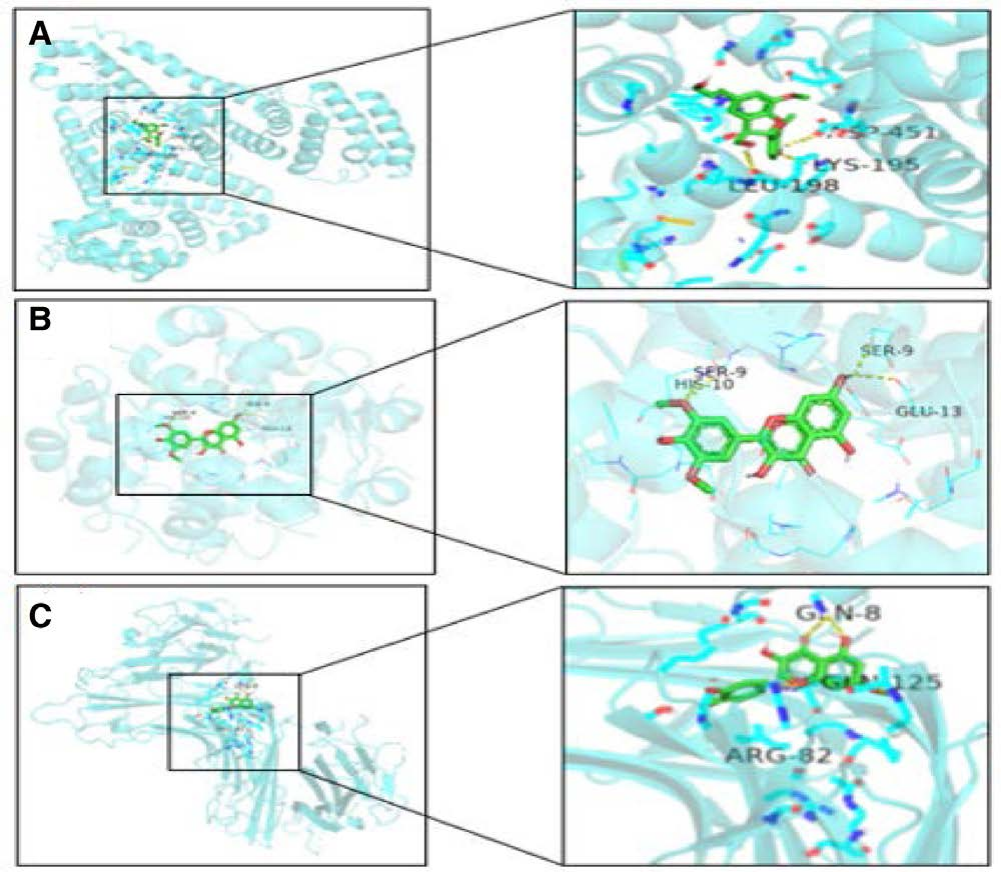
Three-dimensional schematic diagram of the molecular docking model and active site of the main components of p-hydroxybenzaldehyde, carotoside and palmitic acid. (A) The molecular docking of p-hydroxybenzaldehyde and ALB (ASP is aspartic acid, LYS is Lysine, and LEU is leucine). (B) The molecular docking of carotoside and TNF (SER is serine, GLU is glutamic acid and HIS is Histidine). (C) The molecular docking of palmitic acid and INS (GLN is glutamine and ARG is arginine).

Molecular docking simulates the interaction between small molecular ligands and bio-macromolecule receptors, which can predict the binding mode and affinity between them and selects the lead drug of the target, it could aid drug development. In this study, the top 3 targets with the highest degree value were selected for molecular docking, the results were shown in Figure 7. According to the Figure 7, the target of smaller binding energy had stronger binding affinity with the target protein. The binding energy of ALB, INS, and TNF was −6.3, −6.49, and −6.67 kcal/mol, so the target could easily bind its corresponding protein. These results can indirectly confirm the regulatory effect of TMDZ capsules on IS targets. At the same time, the above molecular docking results are consistent with the previous network screening results.

## 4. Discussion

IS is a common cardiovascular and cerebrovascular disease, which is one of the main causes of death and disability, it is a global public health problem. IS has a great impact on the life and health of patients and has caused a large economic burden to the country.^[25–27]^ In recent years, TMDZ capsules have been widely used for the treatment of IS. However, the underlying mechanism of action is still poorly understood when TMDZ capsules are used to treat IS. Network pharmacology approach was applied to explore the underlying pharmacological mechanism of actions of TMDZ capsules and a total of 130 potential targets related to the treatment of IS were identified in this study. Through these targets, we can identify the main ingredients for the treatment of IS including sitosterol, vanillin, palmitic acid, carotene, gastrodin, p-hydroxybenzaldehyde, citric acid, kaempferol, curcumin, ferulic acid, epicatechin and other ingredients. At present, it has been reported that sitosterol, vanillin, carotene, gastrodin, p-hydroxybenzaldehyde and other components can reduce blood cholesterol, inhibit the formation of cerebral arteriosclerosis, inhibit the excessive accumulation of cholesterol in nerve cells, anti-apoptosis of nerve cells, maintain the homeostasis of neurogliocyte and protect the integrity of blood-brain barrier.^[28–32]^ In the treatment of IS, these components can inhibit the accumulation of cholesterol in nerve cells, regulate the cholesterol transport function of cholesterol transporter NPC1L1 on the cell membrane, reduce the expression levels of Cathepsin B and Caspase-3 and inhibit the apoptosis pathway of Cathepsin B-caspase. kaempferol, curcumin, ferulic acid, epicatechin and other components regulate the phosphorylation level of Akt and Src proteins,^[33]^ prolong the reaction time of PT and APTT,^[34]^ increase the activity of plasma t-PA,^[35]^ decrease the activity of PAI-1,^[36]^ increase the content of intracellular CGMP,^[37]^ antagonistic endothelin,^[38]^ blocking calcium channels,^[39]^ dilating blood vessels,^[40]^ reducing blood viscosity, reducing platelet aggregation,^[41]^ restoring damaged endothelial tissue,^[42]^ enhancing the activity of plasma anticoagulant system,^[43]^ enhancing the activity of fibrinolytic system,^[44]^ improving blood perfusion,^[45]^ thrombolysis, inhibiting thrombosis, activating platelets,^[46]^ reducing the release of platelet inflammatory factors,^[47]^ reducing the density and coverage of platelet accumulation in cerebral thrombosis and other ways to anti-oxidation, anti-inflammation, anti-tumor and neuroprotection and other pharmacological effects.^[42]^ These pharmacological effects can well protect cranial nerve function, reduce brain damage, reduce cerebral infarction volume and neurological score in IS. These components play a good role in the treatment of IS.

GO analysis showed that TMDZ capsule played a therapeutic role in the treatment of IS mainly through ion channels, enzyme reactions and hormone reactions. In the early stage and recovery stage of IS, TMDZ capsule can prevent and treat IS by regulating the levels of ion channels, enzyme levels and oxidoreductase activity. KEGG pathway enrichment analysis showed that TMDZ capsule played a role in the treatment of IS mainly through lipid and atherosclerosis, fluid shear stress and atherosclerosis, pathways of neurodegeneration multiple diseases and other pathways.

Through molecular docking, it can be seen that p-hydroxybenzaldehyde, carotoside and palmitic acid molecules are closely connected with the targets of ALB, TNF and INS, which can be inferred that the main drugs in the TMDZ capsule are gastrodia and eumoides. Its main components play a role in the prevention and treatment of IS by reducing the level of EAA neurotransmitter Glu,^[48]^ promoting endothelial cells to release neurotrophic factors VEGF-A and BDNF,^[49]^ enhancing nerve cell to resistance damage,^[50]^ promoting the proliferation of neural stem cells and regulating cholesterol levels.^[51,52]^ This role is mainly related to lipids, ion channels, atherosclerosis pathways and neurodegeneration pathways. The results showed that TMDZ capsules had the effects of anti-oxidation, reducing blood cholesterol, inhibiting cerebral arteriosclerosis, inhibiting thrombosis, activating platelets and reducing the release of platelet inflammatory factors. Furthermore, the mechanism of action of TMDZ capsules was further explained in the prevention and treatment of IS.

## 5. Conclusion

This study systematically explored the active ingredients of TMDZ capsules and the potential targets and signaling pathways of TMDZ capsules in the treatment of IS. The results showed that the TMDZ capsule was composed of 12 drugs, a total of 59 active compounds and 648 targets. There were 2286 targets for IS, and 130 targets for the common action of TMDZ capsules and IS. TMDZ capsule mainly acts through vanillin, p-hydroxybenzaldehyde, palmitic acid, carotene, quercetin, β-carotene and other components in the treatment of IS. The corresponding targets of these components are ALB, TNF, INS, and so on. It mainly plays a role through lipid and atherosclerosis, fluid shear stress and atherosclerosis, neurodegenerative pathway-multiple diseases and other pathways. In addition, molecular docking successfully verified the tight connection between the active compounds and the IS targets. This study explored the mechanism of action of TMDZ capsules in the treatment of IS by network pharmacology, which provided some reference for the clinical use of TMDZ capsules in the treatment of IS.

## Author contributions

**Data curation:** Fengjiao Yang.

**Formal analysis:** Fengjiao Yang.

**Methodology:** Ya Yan.

**Project administration:** Guangming Wang.

**Resources:** Yun Gu.

**Validation:** Fengjiao Yang.

**Visualization:** Ya Yan, Yun Gu.

**Writing – original draft:** Fengjiao Yang.

**Writing – review & editing:** Guangming Wang.

## References

[1] Jayaraj RL Azimullah S Beiram R. Neuroinflammation: friend and foe for ischemic stroke. J Neuroinflammation. 2019;16:142. 31291966 10.1186/s12974-019-1516-2 PMC6617684

[2] Tao T Liu M Chen M. Natural medicine in neuroprotection for ischemic stroke: challenges and prospective. Pharmacol Ther. 2020;216:107695. 32998014 10.1016/j.pharmthera.2020.107695

[3] Ortiz de Mendivil A Alcalá-Galiano A Ochoa M. Brainstem stroke: anatomy, clinical and radiological findings. Semin Ultrasound CT MR. 2013;34:131–41. 23522778 10.1053/j.sult.2013.01.004

[4] Sarikaya H Ferro J Arnold M. Stroke prevention – medical and lifestyle measures. Eur Neurol. 2015;73:150–7. 25573327 10.1159/000367652

[5] Paul S Candelario-Jalil E. Emerging neuroprotective strategies for the treatment of ischemic stroke: an overview of clinical and preclinical studies. Exp Neurol. 2021;335:113518. 33144066 10.1016/j.expneurol.2020.113518 PMC7869696

[6] Xu S Lu J Shao A. Glial cells: role of the immune response in ischemic stroke. Front Immunol. 2020;11:294. 32174916 10.3389/fimmu.2020.00294 PMC7055422

[7] Barthels D Das H. Current advances in ischemic stroke research and therapies. Biochim Biophys Acta Mol Basis Dis. 2020;1866:165260. 31699365 10.1016/j.bbadis.2018.09.012 PMC6981280

[8] Li DH Su YF Sun CX. A network pharmacology-based identification study on the mechanism of Xiao-Xu-Ming Decoction for cerebral ischemic stroke. Evid Based Complement Alternat Med. 2020;2020:2507074. 33133212 10.1155/2020/2507074 PMC7593742

[9] Dong R Huang R Shi X. Exploration of the mechanism of luteolin against ischemic stroke based on network pharmacology, molecular docking and experimental verification. Bioengineered. 2021;12:12274–93. 34898370 10.1080/21655979.2021.2006966 PMC8810201

[10] Wang Y Xiao G He S. Protection against acute cerebral ischemia/reperfusion injury by QiShenYiQi via neuroinflammatory network mobilization. Biomed Pharmacother. 2020;125:109945. 32028240 10.1016/j.biopha.2020.109945

[11] Hong LZ Gu WW Ni Y. Postischemic long-term treatment with Qiangli Tianma Duzhong capsule improves brain functional recovery via the improvement of hemorrheology and the inhibition of platelet aggregation in a rat model of focal cerebral ischemia. Evid Based Complement Alternat Med. 2013;2013:795365. 24319485 10.1155/2013/795365 PMC3830819

[12] Tuo QZ Zhang ST Lei P. Mechanisms of neuronal cell death in ischemic stroke and their therapeutic implications. Med Res Rev. 2022;42:259–305. 33957000 10.1002/med.21817

[13] Zhao J Lv C Wu Q. Computational systems pharmacology reveals an antiplatelet and neuroprotective mechanism of Deng-Zhan-Xi-Xin injection in the treatment of ischemic stroke. Pharmacol Res. 2019;147:104365. 31348992 10.1016/j.phrs.2019.104365

[14] Liu K Tao X Su J. Network pharmacology and molecular docking reveal the effective substances and active mechanisms of Dalbergia Odoriferain protecting against ischemic stroke. PLoS One. 2021;16:e0255736. 34582494 10.1371/journal.pone.0255736 PMC8478192

[15] Wang Y Chu F Lin J. Erianin, the main active ingredient of Dendrobium chrysotoxum Lindl, inhibits precancerous lesions of gastric cancer (PLGC) through suppression of the HRAS-PI3K-AKT signaling pathway as revealed by network pharmacology and in vitro experimental verification. J Ethnopharmacol. 2021;279:114399. 34246740 10.1016/j.jep.2021.114399

[16] Liu N Liu C Yang Y. Xiao-Xu-Ming decoction prevented hemorrhagic transformation induced by acute hyperglycemia through inhibiting AGE-RAGE-mediated neuroinflammation. Pharmacol Res. 2021;169:105650. 33964468 10.1016/j.phrs.2021.105650

[17] Bai LL Chen H Zhou P. Identification of Tumor Necrosis Factor-Alpha (TNF-α) inhibitor in rheumatoid arthritis using network pharmacology and molecular docking. Front Pharmacol. 2021;12:690118. 34093213 10.3389/fphar.2021.690118 PMC8175775

[18] Ma C Wang X Xu T. An integrative pharmacology-based analysis of refined qingkailing injection against cerebral ischemic stroke: a novel combination of baicalin, geniposide, cholic acid, and hyodeoxycholic acid. Front Pharmacol. 2020;11:519. 32457601 10.3389/fphar.2020.00519 PMC7227481

[19] Zhang C Liao Y Liu L. A network pharmacology approach to investigate the active compounds and mechanisms of musk for ischemic stroke. Evid Based Complement Alternat Med. 2020;2020:4063180. 32714405 10.1155/2020/4063180 PMC7354650

[20] Yang Y He Y Wei X. Network pharmacology and molecular docking-based mechanism study to reveal the protective effect of salvianolic acid C in a rat model of ischemic stroke. Front Pharmacol. 2022;12:799448. 35153756 10.3389/fphar.2021.799448 PMC8828947

[21] Wang Y Zhang Y Wang Y. Using network pharmacology and molecular docking to explore the mechanism of Shan Ci Gu (Cremastra appendiculata) against non-small cell lung cancer. Front Chem. 2021;9:682862. 34178945 10.3389/fchem.2021.682862 PMC8220148

[22] Szklarczyk D Morris JH Cook H. The STRING database in 2017: quality-controlled protein-protein association networks, made broadly accessible. Nucleic Acids Res. 2017;45:D362–8. 27924014 10.1093/nar/gkw937 PMC5210637

[23] Zhang L Han L Wang X. Exploring the mechanisms underlying the therapeutic effect of Salvia miltiorrhiza in diabetic nephropathy using network pharmacology and molecular docking. Biosci Rep. 2021;41:BSR20203520. 10.1042/BSR20203520 PMC8209169 33634308

[24] Tong ZQ Tong JR Zhu YL. Revealing the common mechanisms of scutellarin in angina pectoris and ischemic stroke treatment via a network pharmacology approach. Chin J Integr Med. 2021;27:62–9. 32447519 10.1007/s11655-020-2716-4

[25] Barfejani AH Jafarvand M Seyedsaadat SM. Donepezil in the treatment of ischemic stroke: review and future perspective. Life Sci. 2020;263:118575. 33058916 10.1016/j.lfs.2020.118575

[26] Liu J Guo ZN Yan XL. Crosstalk between autophagy and ferroptosis and its putative role in ischemic stroke. Front Cell Neurosci. 2020;14:577403. 33132849 10.3389/fncel.2020.577403 PMC7566169

[27] Jian Z Liu R Zhu X. The involvement and therapy target of immune cells after ischemic stroke. Front Immunol. 2019;10:2167. 31572378 10.3389/fimmu.2019.02167 PMC6749156

[28] Jiang LH Yuan XL Yang NY. Daucosterol protects neurons against oxygen-glucose deprivation/reperfusion-mediated injury by activating IGF1 signaling pathway. J Steroid Biochem Mol Biol. 2015;152:45–52. 25864625 10.1016/j.jsbmb.2015.04.007

[29] Wang P Li C Liao G. Vanillin attenuates proinflammatory factors in a tMCAO mouse model via inhibition of TLR4/NF-kB signaling pathway. Neuroscience. 2022;491:65–74. 35276304 10.1016/j.neuroscience.2022.03.003

[30] Zhao TY Li Z Lei S. Associations for *BCO2, PCSK9*, and *TR1B1* polymorphism and lifestyle factors with ischemic stroke: a nested case-control study. Yonsei Med J. 2019;60:659–66. 31250580 10.3349/ymj.2019.60.7.659 PMC6597471

[31] Zhu T Wang L Wang LP. Therapeutic targets of neuroprotection and neurorestoration in ischemic stroke: applications for natural compounds from medicinal herbs. Biomed Pharmacother. 2022;148:112719. 35168073 10.1016/j.biopha.2022.112719

[32] Xiao T Yang L Chen P. Para-hydroxybenzaldehyde against transient focal cerebral ischemia in rats via mitochondrial preservation. Exp Ther Med. 2022;24:716. 36382091 10.3892/etm.2022.11652 PMC9638521

[33] Abeyrathna P Su Y. The critical role of Akt in cardiovascular function. Vascul Pharmacol. 2015;74:38–48. 26025205 10.1016/j.vph.2015.05.008 PMC4659756

[34] Zheng X Liu H Ma M. Anti-thrombotic activity of phenolic acids obtained from Salvia miltiorrhiza f. alba in TNF-α-stimulated endothelial cells via the NF-κB/JNK/p38 MAPK signaling pathway. Arch Pharm Res. 2021;44:427–38. 33847919 10.1007/s12272-021-01325-7

[35] Kolodziejczyk-Czepas J Ponczek MB Nowak P. Peroxynitrite and fibrinolytic system-The effects of peroxynitrite on t-PA-induced plasmin activity. Int J Biol Macromol. 2015;81:212–9. 26234576 10.1016/j.ijbiomac.2015.07.059

[36] Zhang S Cao Y Du J. Neutrophil extracellular traps contribute to tissue plasminogen activator resistance in acute ischemic stroke. FASEB J. 2021;35:e21835. 34449927 10.1096/fj.202100471RR

[37] Bhat A Tan V Heng B. Papaverine, a phosphodiesterase 10a inhibitor, ameliorates quinolinic acid-induced synaptotoxicity in human cortical neurons. Neurotox Res. 2021;39:1238–50. 33914237 10.1007/s12640-021-00368-4

[38] Wanderer S Mrosek J Vatter H. Crosstalk between the angiotensin and endothelin system in the cerebrovasculature after experimental induced subarachnoid hemorrhage. Neurosurg Rev. 2018;41:539–48. 28756589 10.1007/s10143-017-0887-z

[39] Wang Q Liu N Ni YS. TRPM2 in ischemic stroke: structure, molecular mechanisms, and drug intervention. Channels (Austin). 2021;15:136–54. 33455532 10.1080/19336950.2020.1870088 PMC7833771

[40] Tuttolomondo A Daidone M Pinto A. Endothelial dysfunction and inflammation in ischemic stroke pathogenesis. Curr Pharm Des. 2020;26:4209–19. 32303167 10.2174/1381612826666200417154126

[41] Chen R Wu P Cai Z. Puerariae Lobatae Radix with chuanxiong Rhizoma for treatment of cerebral ischemic stroke by remodeling gut microbiota to regulate the brain-gut barriers. J Nutr Biochem. 2019;65:101–14. 30710886 10.1016/j.jnutbio.2018.12.004

[42] Zong X Li Y Liu C. Theta-burst transcranial magnetic stimulation promotes stroke recovery by vascular protection and neovascularization. Theranostics. 2020;10:12090–110. 33204331 10.7150/thno.51573 PMC7667689

[43] Ząbczyk M Natorska J Undas A. Fibrin clot properties in atherosclerotic vascular disease: from pathophysiology to clinical outcomes. J Clin Med. 2021;10:2999. 34279484 10.3390/jcm10132999 PMC8268932

[44] Henderson SJ Weitz JI Kim PY. Fibrinolysis: strategies to enhance the treatment of acute ischemic stroke. J Thromb Haemost. 2018;16:1932–40. 29953716 10.1111/jth.14215

[45] Campbell BC Mitchell PJ Kleinig TJ.; EXTEND-IA Investigators. Endovascular therapy for ischemic stroke with perfusion-imaging selection. N Engl J Med. 2015;372:1009–18. 25671797 10.1056/NEJMoa1414792

[46] Zhang L Ma J Yang F. Neuroprotective effects of quercetin on ischemic stroke: a literature review. Front Pharmacol. 2022;13:854249. 35662707 10.3389/fphar.2022.854249 PMC9158527

[47] Li X Huang L Liu G. Ginkgo diterpene lactones inhibit cerebral ischemia/reperfusion induced inflammatory response in astrocytes via TLR4/NF-κB pathway in rats. J Ethnopharmacol. 2020;249:112365. 31678414 10.1016/j.jep.2019.112365

[48] Aronica E Condorelli DF Nicoletti F. Metabotropic glutamate receptors in cultured cerebellar granule cells: developmental profile. J Neurochem. 1993;60:559–65. 7678285 10.1111/j.1471-4159.1993.tb03185.x

[49] Ma T Wang Y Qi F. The effect of synthetic oxygen carrier-enriched fibrin hydrogel on Schwann cells under hypoxia condition in vitro. Biomaterials. 2013;34:10016–27. 24095255 10.1016/j.biomaterials.2013.09.047

[50] Li SR Song YJ Deng R. Mallotus oblongifolius extracts ameliorate ischemic nerve damage by increasing endogenous neural stem cell proliferation through the Wnt/β-catenin signaling pathway. Food Funct. 2020;11:1027–36. 31819940 10.1039/c9fo01790a

[51] Liu Y Yang J Che X. Agonistic analog of growth hormone-releasing hormone promotes neurofunctional recovery and neural regeneration in ischemic stroke. Proc Natl Acad Sci USA. 2021;118:e2109600118. 34782465 10.1073/pnas.2109600118 PMC8617525

[52] Zhang L Song K Zhu M. Proprotein convertase subtilisin/kexin type 9 (PCSK9) in lipid metabolism, atherosclerosis and ischemic stroke. Int J Neurosci. 2016;126:675–80. 26040332 10.3109/00207454.2015.1057636

